# Men’s Preferences for Exiting Abdominal Aortic Aneurysm Surveillance: A Deliberative Engagement Session Study

**DOI:** 10.2147/PPA.S554870

**Published:** 2026-01-10

**Authors:** Jane Hughes, Elizabeth Lumley, Alan Elstone, Jonathan Michaels, Akhtar Nasim, Stephen Christopher Radley, Phil Shackley, Gerard Stansby, Emily Wood, Niall MacGregor-Smith, Jo Hall, Alicia O’Cathain

**Affiliations:** 1 School of Medicine and Population Health, Sheffield Centre for Health and Related Research (SCHARR),The University of Sheffield, Sheffield, UK; 2 Vascular Surgery Department, University Hospitals Plymouth NHS Trust, Plymouth, UK; 3 Sheffield Vascular Institute, Northern General Hospital, Sheffield, UK; 4 Department of Vascular Surgery, University of Newcastle and Newcastle Upon Tyne Hospitals NHS Foundation Trust, Newcastle upon Tyne, UK; 5 Department of Clinical Psychology, Derbyshire Community Health Services NHS Foundation Trust, Matlock, UK

**Keywords:** abdominal aortic aneurysm, vascular, screening and surveillance, deliberative engagement session

## Abstract

**Purpose:**

The national screening programme guidance for Abdominal Aortic Aneurysm (AAA) in England states that men with small AAAs will exit surveillance after 15 years. This study explored the informed preferences of men for AAA surveillance.

**Patients and Methods:**

A Deliberative Engagement Session was conducted in two workshops comprising 30 men in AAA surveillance and six of their family members. The workshops consisted of measurement of men’s knowledge and preferences pre- and post-deliberation, presentations by experts, and deliberation by men and their family members, in terms of knowledge sharing and discussion.

**Results:**

Before deliberation, only two of the 30 men in the session were aware of the existence of an exit strategy from AAA surveillance, and their knowledge levels about AAA were poor. Post-deliberation, knowledge levels increased from a median score of 6 (IQR 4 to 7) to a median score of 8 (IQR 8 to 9) correct answers to 11 multiple-choice questions about AAA and AAA screening (p < 0.001). Men in the session identified rate of growth of AAA, size of AAA, health issues that may make surgery risky, and the views of healthcare professionals as important factors to consider in any exit strategy. Most men in the session preferred a strategy whereby they were not discharged from surveillance but had longer intervals between scans (two yearly rather than yearly). Discussion revealed the importance to men and family members of the reassurance surveillance offered to them. In terms of how decisions should be made regarding exit, men in this session wanted to have estimates of the risks of different options, to discuss exit with a Nurse Specialist, and that the patient should decide.

**Conclusion:**

Based on informed preferences, men in this Deliberative Engagement Session preferred longer intervals between scans rather than exiting surveillance because of the reassurance offered by surveillance.

## Background

An Abdominal Aortic Aneurysm (AAA) is a swelling or bulge in the aorta measuring over 3 cm in size. AAAs can increase in size over time and may rupture.^1^ If a rupture occurs, this causes internal bleeding that is often fatal. Rupture is uncommon until the AAA reaches 5.5 cm or greater.^1^ Approximately 3000 people are registered as dying each year in England and Wales following a rupture.^1^ Ruptured abdominal aortic aneurysms have a mortality rate of approximately 80%. Some countries operate national screening programmes for AAA, including Sweden and the four nations of the United Kingdom. In England, the National Health Service (NHS) AAA Screening programme offers screening to all men in their 65^th^ year, when risk of AAA becomes more prevalent. Only men are screened because they are six times more likely to have an AAA than women.^2^ Men found to have AAA enter surveillance rather than have treatment. They receive regular scans annually, or quarterly, depending on the AAA size, to monitor AAA growth.^3^ The NHS screening programme offers each man a consultation with a specialist nurse when entering surveillance or moving from annual to quarterly surveillance. If the AAA reaches 5.5cm or over, they are referred for consideration for treatment such as surgery.

When the NHS AAA screening programme was established, it included guidance for men to be discharged from surveillance (an “Exit Strategy”). Men with small AAAs (between 3 cm and 4.4 cm in diameter) after 15 years would be discharged from surveillance, that is, when they reached 80 years of age.^4^ Given that the AAA screening programme became national in 2013, this guidance will affect all men in AAA surveillance in England from 2028 onwards depending on when they entered surveillance. There has been a reduction in AAA prevalence in the last two decades, but screening is still considered to be cost-effective^5^. The rationale for the Exit Strategy is not documented but is likely to be related to there being little population health benefit in continuing to scan men who are at low risk of AAA rupture. There may also be an implicit recognition that as men age, they may not be healthy enough for surgical treatment. This is an important topic of debate amongst vascular surgeons. Some vascular surgeons have recommended an assessment of fitness for surgery rather than basing decisions on age.^6^ Others have recommended that frailty might be a better determinant than age.^7^,^8^ Other research has noted the lack of guidelines around surveillance in people over 80 years of age with small aneurysms.^9^ Recent international guidelines address exit from AAA surveillance by stating that Patients with small abdominal aortic aneurysms who are either not expected to reach the diameter threshold for repair within their life expectancy, or are unfit for repair, or prefer conservative management, should be considered for discontinuation of surveillance (p.213).^5^

There is an evidence gap in terms of understanding men’s preferences for exiting surveillance. Eliciting patient preferences is an important part of any decision on how any health programme is provided.^10–12^ As part of a wider study of men’s experiences of AAA surveillance, we aimed to explore men’s preferences around exiting AAA surveillance.

## Methods

### The Wider Study

The wider study had two parts (https://www.sheffield.ac.uk/scharr/research/centres/hcru/pcaaas): one part focused on the psychosocial consequences of screening-detected AAA and the other part on the exit strategy.^13^,^14^, When researching the exit strategy, a workshop was held to explore clinicians’ views, men and family members were interviewed, and a telephone survey of 500 men’s preferences was planned. It became clear during qualitative interviews with men that they had never heard of the current plan for exiting surveillance.^13^ Only one out of 27 participants interviewed were aware that surveillance might end. It was also apparent in these interviews that men’s knowledge about AAA and the screening programme was poor. This severely compromised plans for the next stage - the telephone survey of 500 men in AAA surveillance – because of concerns that the survey would be based on uninformed views. The survey was replaced with a Deliberative Engagement Session DES), a method that aims to improve participants’ knowledge base before seeking preferences.^15^ The Deliberative Engagement Session is reported here.

### Deliberative Engagement Session

Deliberative Engagement Session is used to elicit informed preferences from patients and other stakeholders on policy issues.^15^ It is considered to be superior to surveys when stakeholders have limited knowledge about the decision under study, where sophisticated knowledge is required, and where a context may be emotionally charged.^16^ Similar approaches have been used to consider the modernisation of the newborn screening system in the USA, allocation of limited health care resources in the USA, and cancer drugs in Canada.^16–18^ It has recently been used, alongside a national survey, to shape the policy agenda for the NHS in England.^19^ It consists of educational presentations by content experts, facilitated small group discussions, plenary discussions, and pre and post surveys.^15^,^16^ It is a mixed methods approach where the surveys measure change in knowledge and preferences, and qualitative data from the discussion groups highlight the rationale for participants’ expressed preferences. It is described as a cooperative process that allows participants to consider and evaluate the interests and perspectives of their fellow deliberators.^16^ This method is designed to increase the likelihood of eliciting informed, reflective, patient-centred preferences, which may more accurately reflect underlying attitudes.^15^ This deliberative process can provide patient-centred policy recommendations and highlight the rationales underlying them.^16^

## Ethics

Ethics approval was obtained from Wales REC 6 (ref 23/WA/0019).

### The Workshops

There were two five-hour face-to-face workshops in different regions of England (Durham and Plymouth) to ensure diversity of participation and minimise travel for participants. Both workshops were the same in format and content; but with different participants. The workshops were planned in collaboration with the study Patient and Public Involvement panel.

### Patient and Public Involvement Panel (PPI)

One member of the research team – and co-author of the paper – has been diagnosed with an AAA. He actively contributed to the design and delivery of the study as co-applicant on the funding grant and by attending monthly project meetings where decisions were made about how best to conduct the research. The study PPI panel comprised five men who have undergone AAA screening, including four with a diagnosis of AAA. This panel met regularly throughout the project and were invited specifically to review workshop content and advise on the participant invitations, questionnaire and discussion guides for the DES.

### Recruitment

In the part of the wider study exploring the psychosocial consequences of surveillance, 734 men in surveillance, from five AAA screening providers, responded to a postal survey.^14^ Seventy percent agreed to further contact to participate in other parts of the study. The DES sample was selected from this group by focusing on two of the AAA screening providers for geographical diversity All of those who agreed to further contact were invited to participate in the DES. The sample of men who replied included men of different ages, with varied AAA size and growth rates, and from different socio-economic backgrounds. Men were also asked to invite a family member. Members of the PPI panel and staff providing care to men within the AAA surveillance programme were also invited to participate in the discussions.

### Data Collection

All participants gave written informed consent for the workshop to be recorded and transcribed and consented to publication of anonymized responses and direct quotes. Consent included completion of both a pre- and post-deliberation questionnaire. The pre-deliberation questionnaire was completed by men and family members on arrival, before the discussions began. PPI members did not complete the questionnaires due to their prior knowledge and involvement with the research. Then, two vascular surgeons gave short presentations about AAA and the screening programme, and what happens if an AAA reaches 5.5 cm or larger. These presentations were followed by small group discussions facilitated by social science members of the research team. Groups comprised men, family members, a screening staff participant and PPI group members. Next, researchers (social scientists) gave short presentations about how men might make decisions about exiting surveillance, as well as information about alternative exit strategies, which had been developed from results of the first two stages of the exit strategy research study. After further small-group and plenary discussions (again facilitated by social scientists), each man and family member completed the post-deliberation questionnaire. The participants received £50 remuneration and travel expenses.

The pre- and post-deliberation questionnaires were mainly the same: multiple-choice questions to assess men’s knowledge about AAA and the screening programme, views on the importance of a range of factors when developing an exit strategy, and the acceptability of different exit strategies (see Appendix 1). Decision preferences, measures of the value of surveillance as well as perceptions of information available were also collected. There were open questions regarding additional preferred strategies, the most acceptable strategy, and the least acceptable strategy. Participants were asked to identify further factors to be considered in any Exit Strategy [See Appendix 1 for a copy of the post deliberation questionnaire]. The pre-deliberation questionnaire also asked men about sources of information about AAA. The questionnaire was developed by the research team who consisted of vascular surgeons, an AAA specialist nurse, a health economist, social scientists, and PPI members. The questionnaire was reviewed and refined through an iterative process. The list of different exit strategies was devised by the team based on analysis of clinician and men’s views reported in the earlier stages of the wider study. It was not an exhaustive list of potential exit strategies because of the need to ensure the questionnaire was short and easy to complete.

### Analysis

Data from the two workshops were combined. Answers to fixed response questions pre- and post-deliberation were compared using SPSS version 29.0.^20^ For the knowledge questions, the number of correct answers per man was calculated and the median score compared pre- and post-deliberation using the one sample Wilcoxon test. The expectation was that, by providing an opportunity to present detailed AAA-specific information, followed by discussions and consideration of a range of viewpoints, there would be a measurable improvement in knowledge levels over time. Therefore, preferences expressed post-deliberation would be informed preferences. For the remaining questions, answers were 5 point Likert scales ranging from “strongly agree” to “strongly disagree”. Change over time in preferences were compared by creating dichotomous variables by combining the first two categories in each Likert scale into one group and the last three categories into a second group. Then, McNemar’s test was used to compare change over time within the DES participants. Some men did not answer a specific question on both questionnaires so were excluded from the analysis of that question. This analysis was based only on men’s answers, presented in tables. They are presented as the preferences for participants in the DES, and how they changed over time. They are not presented as representative of the views of all men in AAA surveillance, because the DES participants were likely not representative of this population. A sensitivity analysis was undertaken, including family members’ preferences, which showed similar results (see Appendix 1).

Discussions were audio-recorded, transcribed verbatim, and combined with free-text answers on the questionnaires. Content analysis was used to identify rationales for holding preferences and reasons for changing preferences over time.^21^

## Results

### Participants

A total of 300 men were invited to participate. In total, 36 men and their families participated in the DES workshops. Ten men and two family members attended the first workshop and twenty men and four family members attended the second workshop. The workshops took place in different regions to ensure diversity of participation and minimise travel. The median age of the men was 74 (IQR 71 to 76). Nine men (30%) were from the two most socially deprived quintiles in England.^22^ Family members were spouses and adult children. Two members of the PPI panel also participated in the discussions, as well as a screening technician and specialist nurse, but did not complete the questionnaires.

## Knowledge About AAA and Screening

The questionnaires showed that men’s knowledge of AAA and screening increased over time, from a median score of 6.0 (IQR 4–7) correct answers to median score of 8(IQR 8–9) correct answers out of the 11 questions (p = 0.001). Pre-deliberation, only two men (7%) correctly answered the question about exiting surveillance (Q9 in Table 1). Following the presentation and discussion about exiting surveillance, all of the men got the exiting surveillance question right post-deliberation. Pre-deliberation, men’s knowledge was also poor about Q5 (who screening is offered to) with only 20% ticking the correct answer, and Q7 (frequency of screening for medium AAA) with 17% answering correctly (see Table 1). Knowledge appeared to improve for most questions and several of these changes were statistically significant. This indicated that men were offering more informed preferences about an Exit strategy post-deliberation than pre-deliberation.

**Table 1:** Change in the Percentage of Men in the DES Answering Knowledge Questions Correctly (N = 30 Men)

Questions Asked	Pre-Deliberation N (%)	Post-Deliberation N (%)	Change (Based on Those Who Completed Both Pre and Post Deliberation) N (%)
Q1 What is AAA	16 (53%)	23 (77%)	7 (24%)+
Q2 Diameter of a normal AAA	23 (77%)	26 (87%)	3 (10%)
Q3 Definition of a small AAA	16 (53%)	19 (63%)	3 (10%)
Q4 Definition of a medium AAA	13 (43%)	20 (67%)	7 (24%)+
Q5 Who screening is offered to	6 (20%)	6 (20%)	0 (0%)
Q6 Frequency of scanning for small AAA	24 (80%)	29 (97%)	5 (17%)
Q7 Frequency of scanning for medium AAA	5 (17%)	18 (60%)	13 (43%)+
Q8 How is screening carried out	29 (97%)	30 (100%)	1 (3%)
Q9 Exiting surveillance	2 (7%)	30 (100%)	28 (93%)+
Q10 Plan when AAA becomes large	21 (70%)	30 (100%)	9 (30%)+
Q11 Reason for surveillance rather than surgery	24 (80%)	24 (80%)	0 (0%)

**Notes**: +p < 0.05 McNemar’s test.

In discussions, many of the men described how little they knew about AAA and the screening programme, and how they had unanswered questions. They expressed limited understanding of issues such as whether AAA might be hereditary, symptoms to be aware of, and lifestyle changes that might be beneficial or not. One participant commented,

The link to fitness and how important fitness is, that’s reaffirmed it today. In respect of this now [lifestyle discussion], I think I need to lose a few pounds”. (DES participant, Durham)

They also described how much they had learned from the workshop presentations. For example:

I’ve actually learned more this morning in an hour than I have done by going to these screening sessions for ten years. (DES participant, Durham)

## Information About AAA and AAA Screening

On the pre-deliberation questionnaire, most men reported that they had received or found some information about AAA and screening in a written format or through discussions with healthcare staff. Some had accessed information on the Internet, but a fairly high percentage of men in the DES (43%) had not used online resources at all (see Table 2). For both written information and discussions with healthcare staff, 70% of men in the DES reported this to be extremely or very helpful.

**Table 2:** Percentage of Men in the DES Finding Sources of Information About AAA Helpful Pre-Deliberation (N = 30 Men)

	Extremely Helpful	Very Helpful	Slightly Helpful	Not at All Helpful	Not Used This
Written information like leaflets	9 (30%)	12 (40%)	8 (27%)	0 (0%)	1 (3%)
Online information like websites	4 (13%)	6 (20%)	6 (20%)	1 (3%)	13 (43%)
Discussions with health care staff	12 (40%)	9 (30%)	6 (20%)	0 (0%)	3 (10%)

In the discussions, some men expressed a preference for talking to healthcare staff over written information.

Sometimes you get a pamphlet, it doesn’t make an awful lot of sense. You actually really need somebody to sit down and say to you, right, this is what it means, this is what it does, this is what’s going to help it. (DES participant, Plymouth)

## Factors to Consider Within an Exit Strategy

On the questionnaires, when asked about the factors that were important to consider when developing an exit strategy, men were asked to rate 11 factors from “extremely important” to “not at all important” on a five-point Likert scale. The percentages of men scoring each factor as “extremely” or “very important” pre- and post-deliberation were compared (Table 3). In the table, the factors are ordered by men’s scores post-deliberation because these were based on more informed views. The three highest scoring factors post-deliberation were related to AAA and health: rate of growth of AAA, size of AAA, and health problems that can make future surgery risky. The three lowest scoring factors post-deliberation were related to screening-related anxiety and convenience of attending for scans. It was interesting that these views did not change much over time in the DES participants. The exception was the item about age. Pre-deliberation 53% of men in the DES rated age as extremely or very important and post-deliberation only 30% of men scored this factor as extremely or very important, although this was not statistically significant.

**Table 3:** Percentage of Men in the DES Rating Factors Extremely or Very Important Pre- and Post-Deliberation (N = 30 Men)

Factor	Pre-Deliberation	Post-Deliberation
Rate of growth of AAA	30 (100%)	30 (100%)
Size of AAA	29 (97%)	30 (100%)
Health issues that may make surgery risky	27 (90%)	28 (93%)
The views of healthcare professionals	27 (90%)	28 (93%)
What people and their family prefer	20 (67%)	22 (73%)
Reassurance provided by a scan	27 (90%)	26 (87%)
Number of years in surveillance	16 (53%)	12 (40%)
Age	16 (53%)	9 (30%)
Difficulty attending for scans	7 (23%)	7 (23%)
Cost to the NHS	7 (23%)	7 (23%)
Worry caused by being in surveillance	7 (23%)	6 (20%)

In the discussions, men described how the existence of an Exit strategy was a surprise to them.

This is the first time that I knew that there was an exit strategy. I was led to believe that I was in this for life. (DES participant, Durham)

In the discussions, some men were very unhappy about the use of age as a factor in decision-making. Some felt it was not acceptable to base decisions about exiting surveillance on age because many older people have a good quality of life. They felt that using age as a determining factor for exit indicated the lack of value given to older people in society. It is possible that these strongly expressed views about age affected the post-deliberation answers given by other men:

At 80, you’re no value to society, you’re no value to anybody else, go away and die. (DES participant, Plymouth)

Discussions about factors to consider within an exit strategy also revealed the high value men, and their families, placed on reassurance they gained from remaining in surveillance:

I initially accepted the screening for reassurance. Maybe I didn’t want to have an aneurysm like. Now I go for reassurance that the aneurysm is not growing. (DES participant, Durham)

## Acceptability of Different Exit Strategies

Men were presented with nine potential exit strategies and asked to rate them on a five point Likert scale from “very acceptable” to “very unacceptable” (see Table 4). These have been ordered in Table 4 by the most acceptable in the post-deliberative assessment. The three most acceptable exit strategies for the men in the DES were: longer time between scans rather than exit, a discussion with a specialist nurse if health becomes worse, and an assessment for suitability for surgery at 4.5 cm. The two least acceptable were most similar to the current exit strategy. Again, there was little change in scores over time. The exception was that less men found the current exit strategy acceptable post-deliberation (see ES1 in Table 4), although this was not statistically significant (p < 0.065). When asked to select the strategy most acceptable to them post-deliberation, ES9 (small AAAs could have a longer scan interval after five years) was selected by a large proportion of participants (n = 12/30 (40%)). When asked to select the strategy least acceptable to them, ES6 (meeting with staff every year to discuss whether to continue surveillance) was chosen most often.

**Table 4:** Percentage of Men in the DES Rating Exit Strategies Very Acceptable or Acceptable (N = 30)

	Exit Strategy (Short Description)	Pre-Deliberation N (%)	Post-Deliberation N (%)
ES9	If, after 5 years in surveillance, a man’s AAA is not growing or is growing very slowly, he can choose to have a longer interval between scans (for example, have a scan every 2 years). However, if the rate of growth of his AAA increases, he can return to having more regular scans	27 (90%)	27 (90%)
ES7	If a man’s health becomes worse while in surveillance, this will trigger a discussion with a specialist nurse about whether it is appropriate for him to continue in surveillance	24 (80%)	25 (83%)
ES5	If a man’s AAA reaches the size of 4.5cm, he would have an assessment to see if he would be suitable for surgery should he ever need it. Men found to be unsuitable for surgery (due to not being fit/healthy enough or because the aneurysm is too extensive) would be given a choice of whether or not to stay in the surveillance programme	21 (70%)	23 (77%)
ES8	If, after 5 years in surveillance, men with a small AAA that is not growing or is growing very slowly, can choose to exit surveillance. However, they have the option of re-entering the surveillance programme in the future if they have any concerns	26 (87%)	23 (77%)
ES 4	After being in surveillance for 10 years, all men in the surveillance programme would have an assessment to see if they would be suitable for surgery should they ever need it. Men found to be unsuitable for surgery (due to not being fit/healthy enough or because the aneurysm is too extensive) would be given a choice of whether or not to stay in the surveillance programme	21 (70%)	20 (67%)
ES 3	When men are first diagnosed with an AAA, before entering the surveillance programme they would have an assessment to see if they would be suitable for surgery should they ever need it. Men found to be unsuitable for surgery (due to not being fit/healthy enough or because the aneurysm is too extensive) would be given a choice of whether or not to go into the surveillance programme.	15 (50%)	18 (60%)
ES6	Each year, when attending for a scan, all men in surveillance would meet with a health care professional to discuss whether it is appropriate for them to continue in surveillance	22 (73%)	16 (53%)
ES 1	Men in surveillance with a small AAA would be discharged from the programme after 15 yearly scans (ie age 80 for those first scanned at 65 years old)	20 (67%)	13 (43%)
ES 2	Men in surveillance with a medium AAA that is not growing or is growing very slowly would be discharged from the programme after 15 years of scans (ie aged 80 for those first scanned at 65 years old).	12 (40%)	11 (37%)

In the discussions, some men and family members were curious about why there was an exit strategy at all. The presentation was clear that the rationale was not based on the need to make cost savings but rather based on there being a low risk of rupture for small AAAs that had not grown over 15 years. Even so, some men were concerned that the need to reduce the costs of surveillance was driving the existence of the exit strategy. These men had very strong views that costs should not be considered when developing an exit strategy policy:

I Think That Cost Should Be Taken Out of the Equation Totally, (DES Participant, Durham)

Other men and family members expressed awareness of the limited resources within the NHS and viewed increasing the time between each scan rather than leaving surveillance as a strategy that considered both men’s desire for reassurance and costs to the NHS:

I also think in five years, if nothing’s moved, you should be offered two-yearly. And then at the end of ten years if nothing moved, you should be offered three-yearly; because it’s all about resources, isn’t it? (DES participant, Plymouth)

## How Exit Strategy Decisions Should Be Made

Men were asked to “strongly agree” through to “strongly disagree” on a five point Likert scale about 9 aspects of decision-making around exiting surveillance. The percentage of men strongly agreeing or agreeing with aspects of decision-making are shown in order of agreement post-deliberation in Table 5. Post-deliberation, most men in the DES wanted an estimate of the risk for different options, to discuss the options with a nurse specialist, and decide themselves with family and friends. They did not agree that health professionals should decide, or that costs should be taken into account when considering exit options. Again, there were few changes in preferences over time. There was an increase in men saying that they should decide about exiting or not, and a decrease in men wanting a discussion with their GP. Only the latter was statistically significant (p < 0.039).

**Table 5:** Percentage of Men in the DES Strongly Agreeing or Agreeing with Statements About How Decisions Should Be Made (N = 30 Men)

Aspect	Pre-Deliberation N (%)	Post-Deliberation N (%)
Helpful to have estimate of risks of different options	28 (93%)	29 (97%)
Discuss with Nurse Specialist	25 (83%)	25 (83%)
Patient should decide	19 (63%)	25 (83%)
Family/friends involved in discussion	21 (70%)	22 (73%)
Discuss with vascular surgeon	19 (63%)	15 (50%)
Prefer to make decision myself after being provided with written information	15 (50%)	13 (43%)
Discuss with GP	17 (57%)	9 (30%)+
Cost should be taken into account	6 (20%)	8 (27%)
Doctors and nurses should decide	5 (17%)	3 (10%)

**Notes**: +P < 0.05 McNemars’ Test.

During discussions, men expressed the desire for shared decision-making, that is, a discussion between men (with family members if wished for) and experienced healthcare staff.

A conversation with somebody, it’s probably not the screening centre, good though they are, it’d have to be somebody with a little bit more knowledge. (DES participant, Plymouth)

The men felt that ultimately the decision should be the patient’s but wanted information explained to them. Written information was seen as valuable alongside a conversation with healthcare staff:

But unless you’ve got the information by talking to somebody you won’t be able to make a proper choice, would you, anyway. So, you’ve got to talk to somebody who’s got the know-how, and then you can make a choice. (DES participant, Plymouth)

It was clear from the discussion why some men had changed their minds about the preference for talking to GPs. Some men described how GPs were not likely to know much about AAA, and this may have influenced other men’s views.

## The Value of Being in Surveillance

As described earlier, it was clear from the discussion that men valued reassurance from being regularly scanned. To explore the value of surveillance, particularly in the context where men might be too ill to receive treatment in the future, men were asked three questions about what they valued about being in surveillance (see Table 6). When asked to select between reassurance offered by regular scanning and the possibility of future surgical repair, more men in the DES selected the reassurance of being regularly scanned. Pre-deliberation, 80% of men still valued being in surveillance even if they could not have surgery, but following discussions, fewer men (57%) selected this option although this was not a statistically significant change (p < 0.134).

**Table 6:** Percentage of Men in the DES Valuing Different Aspects of Surveillance (N = 30 Men)

Value About Being in Surveillance	Pre-Deliberation	Post-Deliberation
Value most about surveillance		
Reassurance	18 (60%)	20 (67%)
Future surgery	12 (40%)	10 (33%)
Other	0 (0%)	0 (0%)
Value least about surveillance		
Effort of attending	6 (20%)	6 (20%)
Worry when scanned	12 (40%)	11 (37%)
Other	8 (27%)	9 (30%)
Value being in surveillance even if cannot have surgery		
Yes	24 (80%)	17 (57%)
No	2 (7%)	8 (27%)
Do not know	4 (13%)	5 (17%)

In the discussion, men explained why they had different preferences. Some men explained that they still valued being in surveillance because of the information they would continue to receive about their AAA size, even when they had small AAAs and were unlikely to ever need surgery, or too ill to have surgery. Other men changed their preferences as they listened to the presentations and other participants, softening their stance on needing to remain in surveillance:

I said this morning you know, well there’s no way I’m going to be part of an exit strategy, I want to know what I’m like for as long as I can. But now, hang on, there’s another way to look at this. I’m not going to have surgery (due to small AAA size), so, I still want it (surveillance) but it’s whether it’s still annual or whether you just make it less frequent. (DES participant, Plymouth)

Some men who valued being in surveillance so that they could have surgery if they needed it concluded that exit would be best for those in poor health.

But if they tell you you’re not suitable, we can’t do them (surgical options), there’s no point in carrying on with it. (DES participant, Durham)

## Discussion

### Summary of Findings

Pre-deliberation, very few men in this sample were aware of the existence of an exit strategy and their knowledge about AAA was generally poor. Their knowledge about AAA and AAA screening increased by the end of the workshop, so they could express more informed preferences than before deliberation. Men valued being provided with the opportunity within the DES to gain knowledge and understanding about having an AAA and the screening programme. Most men in this DES identified rate of growth of AAA, size of AAA, health issues that may make surgery risky, and the views of healthcare professionals as important factors to consider in any exit strategy. Most men in this DES preferred a strategy whereby men were not discharged from surveillance but had longer intervals between scans (two yearly rather than yearly). Discussions revealed the importance to men of the reassurance surveillance offered. In terms of how decisions should be made about exit, the top three choices by men in this DES were: to have estimates of risks of different options, to discuss exit with a Nurse Specialist, and that the patient should decide themselves. Interestingly, preferences about exit sometimes changed post-deliberation but largely stayed the same as pre-deliberation.

### Context of Other Research

The views men expressed on the open questions in the questionnaire, and during discussions, were similar to views expressed in a qualitative interview study we undertook as part of the wider study.^13^ Men within that qualitative interview study were less welcoming of an exit strategy than clinicians because they valued the reassurance offered by surveillance, and were concerned about basing decisions on age, and suggested the option of longer periods between scans rather than exit. Some men who participated in these interviews also participated in the DES reported here, but most of the men in the DES had not taken part in the previous interview study. Other studies support the DES findings, specifically around poor knowledge levels of men in AAA surveillance, and desire for an option for longer periods between scans rather than exit from surveillance. Poor AAA knowledge levels of people in AAA surveillance have been identified in other countries.^23^ A study of older patients (>85 years) in the UK found that following shared decision-making discussions with medical staff about exiting AAA surveillance, a majority of participants chose to exit, but some wanted to remain in surveillance with longer intervals between scans. It also found that individual level decision making was seen as key by older patients, but a lack of guidelines about exiting was identified as a concern.^9^ In another UK study, following shared decision-making discussions with a decision aid, men with smaller aneurysms (3 cm-3.4 cm) opted for longer intervals of 24 months between scans.^24^

### Strengths and Limitations

The key strength of this approach was that it allowed identification of informed preferences. This is important given men’s low levels of knowledge about AAA, screening, and the existence of an exit strategy. Men’s knowledge levels improved in this exercise; therefore, their post-deliberation views were more informed than their pre-deliberation views. It is important to consider the potential for bias in this study, because it was not a random sample. Those who attended had some diversity in terms of age, AAA size, and social deprivation, but not ethnic minority status, because all men were White British. Invitees may have declined due to a lack of knowledge of the exit strategy or may have had limited travel options due to limited mobility or comorbidities. The statistics presented here cannot be extrapolated to the entire male population in AAA surveillance. Instead, they describe the preferences of men who attended DES and how these preferences changed in light of deliberations.

### Implications

Policymakers need to consider a wide range of issues when reviewing the current policy on exiting AAA surveillance, such as cost-effectiveness and safety. As outlined earlier, the incidence of AAA prevalence is falling, due to factors such as smoking cessation, and this may reduce the cost-effectiveness of AAA screening in future. Recent guidelines and a review of NHS AAA screening, however, suggest that screening is still currently cost-effective, but consideration may be needed as to how this may evolve in future.^5^,^25^ This may include longer periods between scans for small AAAs. The research reported here can bring the views of men and their families into policy deliberations. This research demonstrates that men in this DES, who had been informed about AAA and had exited AAA surveillance, preferred to accept longer periods between scans than exiting surveillance. The research cannot determine the proportion of men in AAA surveillance who would choose to exit or who would choose to remain in surveillance with longer periods between scans but does show that some men have strong preferences for the latter option. Policymakers could consider this option to address men’s need for reassurance from surveillance. If policymakers decide to continue with an exit from surveillance, then a gentle “letting go” may be required for some men.^26^

This study has implications beyond the national AAA screening programme in England. It is relevant to: people who are in surveillance outside the AAA screening programme in England; men who are in surveillance in national programmes in Scotland, Northern Ireland, and Wales, and people in surveillance for other conditions such as cancer where exit from surveillance is used.^27^,^28^ It may be less relevant to people in AAA surveillance in countries that adhere to the 2024 international guidelines on three year surveillance intervals for small AAAs.^5^

## Conclusions

Based on informed preferences, men in this Deliberative Engagement Session preferred longer intervals between scans rather than exiting surveillance because of the reassurance offered by surveillance. This research could not determine the proportion of men in AAA surveillance who would choose to exit or choose to stay because it was a selective rather than a representative sample of men. Nonetheless, the views described here are useful to consider when national policymakers and clinicians develop guidelines about exiting AAA surveillance.
